# Pessary or surgery for a symptomatic pelvic organ prolapse: the PEOPLE study, a multicentre prospective cohort study

**DOI:** 10.1111/1471-0528.16950

**Published:** 2021-10-28

**Authors:** LR van der Vaart, A Vollebregt, AL Milani, AL Lagro‐Janssen, RG Duijnhoven, J‐PWR Roovers, CH Van der Vaart

**Affiliations:** ^1^ Department of Obstetrics and Gynaecology Amsterdam Reproduction & Development Research Institute Amsterdam UMC University of Amsterdam Amsterdam The Netherlands; ^2^ Department of Obstetrics and Gynaecology Spaarne Gasthuis Hoofddorp The Netherlands; ^3^ Department of Obstetrics and Gynaecology Reinier de Graaf Hospital Delft The Netherlands; ^4^ Department of General Practice/Women's studies Medicine University Medical Centre Radboud Nijmegen The Netherlands; ^5^ Department of Gynaecology Bergman Clinics Amsterdam The Netherlands; ^6^ Department of Obstetrics and Gynaecology UMCU University of Utrecht Utrecht The Netherlands; ^7^ Department of Gynaecology Bergman Clinics Bilthoven The Netherlands

**Keywords:** Global improvement, pelvic organ prolapse, pessary, surgery

## Abstract

**Objective:**

To compare the 24‐month efficacy of pessary or surgery as the primary treatment for symptomatic pelvic organ prolapse (POP).

**Design:**

Multicentre prospective comparative cohort study.

**Setting:**

Twenty‐two Dutch hospitals.

**Population:**

Women referred with symptomatic POP of stage ≥2 and moderate‐to‐severe POP symptoms.

**Methods:**

The primary outcome was subjective improvement at the 24‐month follow‐up according to the Patient Global Impression of Improvement (PGI‐I) scale. Secondary outcomes included improvement in prolapse‐related symptoms measured with the Pelvic Floor Distress Inventory (PFDI‐20), improvement in subjective severeness of symptoms according to the Patient Global Impression of Severity (PGI‐S) scale and crossover between therapies. The primary safety outcome was the occurrence of adverse events.

**Main outcome measure:**

PGI‐I at 24 months.

**Results:**

We included 539 women, with 335 women (62.2%) in the pessary arm and 204 women (37.8%) in the surgery arm. After 24 months, subjective improvement was reported by 134 women (83.8%) in the surgery group compared with 180 women (74.4%) in the pessary group (risk difference 9.4%, 95% CI 1.4–17.3%, *P* < 0.01). Seventy‐nine women (23.6%) switched from pessary to surgery and 22 women (10.8%) in the surgery group underwent additional treatment. Both groups showed a significant reduction in bothersome POP symptoms (*P* ≤ 0.01) and a reduction in the perceived severity of symptoms (*P* ≤ 0.001) compared with the baseline.

**Conclusions:**

Significantly more women in the surgery group reported a subjective improvement after 24 months. Both therapies, however, showed a clinically significant improvement of prolapse symptoms.

**Tweetable abstract:**

Pessary treatment and vaginal surgery are both efficacious in reducing the presence and severity of prolapse symptoms, although the chance of significant improvement is higher following surgery.

## Tweetable abstract

Pessary treatment and vaginal surgery are both efficacious in reducing the presence and severity of prolapse symptoms, although the chance of significant improvement is higher following surgery.

## Introduction

Symptomatic pelvic organ prolapse (POP) is a common problem in women, with an estimated prevalence of 8.3–12.1% and with peak incidence found in women aged 60–69 years.^1^, ^2^, ^3^ Although not life threatening, POP negatively affects the quality of life through micturition and defecatory symptoms, vaginal bulging and sexual disorders.^2^, ^4^, ^5^, ^6^

Several treatment options are available for POP, ranging from conservative measures like lifestyle advice, pelvic floor physiotherapy and pessary therapy, to surgery as a more invasive option.^7^, ^8^ The choice between pessary and surgery depends on both doctor as well as patient preference. A survey showed that 69% of gynaecologists always informed their patients about pessary therapy, 17% sometimes did and 14% never did.^9^

Among patients, treatment goals and choices for therapy can differ. Of women with POP who are treated and untreated, 48% preferred surgery, 36% preferred a pessary and 16% had no preference.^10^ Regarding treatment goals, women opting for surgery or using a pessary reported the same treatment goal, namely to improve their prolapse symptoms.^11^ Beside the positive effects of these treatments in improving POP symptoms, both treatments have disadvantages. Side effects of surgery can include newly reported stress urinary incontinence (9.9%), recurrence of POP (36% over 10 years of follow‐up), newly reported dyspareunia (10%) and a reoperation rate of 17%.^12^, ^13^, ^14^, ^15^ Adverse events with pessary treatment may occur in up to 54% of women and include pessary expulsion, discomfort, pressure ulcer, micturition disorders and vaginal discharge.^16^, ^17^ After 24 months 24.5–36.0% of women stopped using a pessary.^18^, ^19^

Studies directly comparing pessary with surgery for symptomatic POP are scarce, are heterogenous, which complicates any comparison of the findings, are underpowered and have a high loss to follow‐up.^11^, ^20^, ^21^ Moreover, outcomes of pessary therapy have mainly been reported in relation to the (dis‐)continuation of treatment, and to a much lesser extent in terms of symptom relief. This makes recommendations on the best treatment option for the patient for them to make an informed decision speculative. This has been recognised in two recent reviews, which both emphasise the urgent need for comparative studies between pessary and surgery for symptomatic POP.^20^, ^22^, ^23^ The recent National Institute for Health and Care Excellence (NICE) guideline on the management of POP also urges clinicians to generate evidence on long‐term outcomes and patient satisfaction.^24^

The aim of this multicentre prospective cohort study with a 24‐month follow‐up, was to compare efficacy between pessary and surgery in women with symptomatic POP in terms of patient satisfaction.

## Methods

### Study design and participants

The PEOPLE project was initiated to compare the effectiveness of pessary and surgery in women with symptomatic POP, and includes a non‐inferiority randomised controlled trial (RCT) and this observational cohort study. While recruitment for the RCT was continuing, many women expressed strong preferences in treatment choice and, as a consequence, refused to participate in the RCT. Therefore, we set up this observational cohort alongside the RCT. This study was performed in 22 Dutch hospitals. The project was funded by ZonMw, a Dutch organisation for innovation and research in health care and was approved by the Medical Ethical Committee of the University Medical Centre Utrecht (UMCU).

We included women who were referred by their general practitioner (GP) with POP stage ≥2 according to the International Continence Society (ICS) POP‐Q system and moderate to severe POP symptoms, defined as a prolapse domain score of >33 on the original Urinary Distress Inventory.^25^ Exclusion criteria were prior prolapse or incontinence surgery, probability of future childbearing, insufficient knowledge of the Dutch language, comorbidity causing increased surgical risks, major psychiatric illness and prior pessary use.

Women were counselled by their gynaecologist about pessary and surgical treatment according to the Dutch guidelines.^26^ After a week, all women were asked if they were willing to participate in the RCT. If a woman actively opted for one these treatments she was invited for follow‐up in this observational cohort. A follow‐up at 6 weeks, 12 months and 24 months was planned as part of the study protocol, and women were instructed to return to the hospital if they experienced any complaint. If self‐management of a pessary was not possible or not preferred, women were seen at 4‐month intervals for pessary cleaning and vaginal inspection. If women performed self‐management, the frequency of cleaning was left to their personal judgement but was advised to be no less frequently than every 4 months.

Patient data were collected in an electronic case report form (openclinica 3.6; OpenClinica, LLC, Waltham, MA, USA). limesurvey 2.6.7 (forums.limesurvey.org) was used to digitally send out questionnaires and store the responses. All patients gave written informed consent.

### Patient involvement

Two gynaecological patient organisations, the ‘Patienten Gynaecologie Nederland’ and the ‘Stichting Bekkenbodem Patienten’, fully supported the study design.

### Interventions

All participating gynaecologists had fitted at least 100 pessaries and had performed more than 100 surgical POP procedures prior to the start of this study. Supportive as well as occlusive pessaries could be used as both are proven to be effective.^27^ The pessary fitting was considered successful if the patient felt comfortable with the pessary in situ and there was no pessary expulsion.

Surgical intervention consisted of correction of those compartments requiring surgery, at the discretion of the gynaecologist. Anterior or posterior colporrhaphy were considered standard procedures for anterior or posterior vaginal wall prolapse, respectively. For uterine descent, uterine‐preserving techniques like sacrospinous hysteropexy (SH) and the modified Manchester–Fothergill procedure or vaginal hysterectomy were performed.^28^, ^29^, ^30^ All patients received prophylactic antibiotics and thrombosis prophylaxis according to local protocols.

### Outcomes

The primary outcome of this study was subjective improvement at the 24‐month follow‐up, measured with the Patient Global Impression of Improvement (PGI‐I) scale. The PGI‐I is a seven‐point Likert scale that ranks the response to a single question from ‘very much worse’ to ‘very much better’, and is a validated instrument and a true reflection of ‘success’ in women undergoing treatment for POP.^31^, ^32^ Subjective improvement was defined as a response of ‘much better’ or ‘very much better’ on the PGI‐I.^33^

Secondary outcomes included crossover of therapy, subjective severity of symptoms measured with the Patient Global Impression of Severity (PGI‐S) scale and pelvic floor distress symptoms, measured with the Pelvic Floor Distress Inventory (PFDI‐20). The PGI‐S scale is a four‐point Likert scale with the following response options: not severe, mild, moderate or severe. If at follow‐up women scored at least one point less on the PGI‐S, they were considered improved. The PFDI‐20 comprises 20 questions in three subscales assessing the experienced bother of POP on specific prolapse (POPDI‐6), bladder (UDI‐6) and bowel (CRADI‐8) symptoms.^34^ Higher scores in a particular domain represent more bothersome symptoms, the subscale scores vary between 0 and 100, and the total PFDI‐20 score varies between 0 and 300 points.^34^ The PFDI‐20 is recommended by the International Consultation on Incontinence (ICI) guideline, validated in Dutch and responsive to change in women undergoing surgical as well as non‐surgical treatment for POP.^34^, ^35^, ^36^

All patients were asked to complete the PGI‐S and PFDI‐20 at baseline, and at the 12‐ and 24‐month follow‐up, and the PGI‐I at the 12‐ and 24‐month follow‐up.

### Study size

Women were recruited into this cohort until a minimum number of women was reached, equal to the projected sample size of the RCT. One hundred and ninety‐eight women per group would achieve 80% power to reject the null hypothesis that pessary therapy is inferior to surgery, with a one‐sided alpha of 0.05, a non‐inferiority margin of 10% and an incidence of clinically significant improvement in the surgery group of 80%. However, the objective of this cohort was not to study non‐inferiority, which is the objective of the non‐inferiority trial in this project, but to study the general improvement of complaints, adverse outcomes and (dis‐)continuation of treatment over a period of 24 months.

### Statistical analysis

Descriptive statistics are presented. Categorical data are presented as numbers with percentages, continuous data as means with standard deviations (SDs) and ordinal data as medians with interquartile ranges (IQRs). Dichotomous variables were analysed using a chi‐square test, continuous variables were tested using the unpaired‐samples Student’s *t*‐test or with the Mann–Whitney *U*‐test, depending on the distribution of normality. The Mann–Whitney *U*‐test was also used for ordinal variables. To assess differences within groups we used a paired‐samples Student’s *t*‐test for the continuous variables and the Wilcoxon signed rank test for ordinal data.

For the primary outcome, we estimated the risk difference and relative risk with 95% confidence intervals on subjective improvement of the PGI‐I between pessary and surgery at the 24‐month follow‐up. Statistical significance was tested using a chi‐square test. Adjustment for confounding factors in the association between PGI‐I score and therapy was performed using multivariable binomial regression (log‐link for relative risks and identity‐link for risk differences). In this model, baseline characteristics with *P* ≤ 0.1 and a minimum of 15 cases were included.^37^ Adjustment for POP‐Q stage was planned irrespective of the difference between groups as we expect this to influence the subjective improvement. Additionally, multivariable binomial regression was performed comparing women who had surgery, had retained a pessary and had switched from pessary to surgery.

For the secondary outcomes, the risk difference was calculated for the PGI‐S. An effect size (ES) calculation, dividing the mean difference by the standard deviation of the difference, was performed for the changes from baseline in PFDI‐20 scores within groups in order to assess the strength of the effect, which is generally considered to be a measure of the clinical relevance.^38^ In general, an ES of 0.8, 0.5 and 0.2 represents a large, medium and small effect size, respectively.^39^

Discontinuation of initial treatment, reoperation, switching from pessary to surgery or additional use of a pessary by women who had already undergone surgery was analysed as a time‐to‐event outcome using a Kaplan–Meier graph and log‐rank test.

spss statistics 26 (IBM, Armonk, NY, USA) and sas (SAS Institute, Cary, NC, USA) were used for statistical analysis.

## Results

### Study population

The recruitment period was between February 2016 and December 2017. Figure S1 shows the flow chart of the study population. A total of 539 women were recruited from 22 centres, with 335 women (62.2%) in the pessary group and 204 women (37.8%) in the surgery group. Data on the primary outcome at 24 months were available for 242 women in the pessary arm and for 160 women in the surgery arm. Baseline characteristics are shown in Table 1. Women in the surgery group were significantly younger, premenopausal, had a higher body mass index (BMI) and more often had a history of caesarean section. With respect to POP‐specific characteristics, significantly more women in the surgery group reported ‘severe’ symptoms on the PGI‐S as well as a higher mean score on the PFDI‐20 subscales and total score.

**Table 1:** Baseline characteristics

Baseline characteristic	Pessary group *n* = 335	Surgery group *n* = 204	*P*
Age	62.8 (±9.6)	59.3 (±9.6)	**<0.001** ^a^
BMI (kg/m^2^)	24.5 (22.9–26.6)	25.4 (23.3–28.6)	**0.001** ^b^
Smoking	45 (13.5%)	30 (14.9%)	0.67^c^
History of gynaecological surgery	55 (16.5%)	42 (20.6%)	0.23^c^
Diabetes	14 (4.2%)	12 (5.9%)	0.38^c^
Chronic pulmonary disease	16 (4.8%)	12 (5.9%)	0.58^c^
Family history of prolapse	145 (43.4%)	105 (51.7%)	0.06^c^
Antidepressant use	16 (4.8%)	11 (5.4%)	0.76^c^
Parity	2.0 (2–3)	2.0 (2–3)	0.76^b^
Mode of delivery			
Caesarean section	7 (2.1%)	12 (5.9%)	**0.02** ^c^
Vacuum‐assisted delivery	29 (9.9%)	12 (6.9%)	0.27^c^
Forceps delivery	11 (3.8%)	13 (7.6%)	0.08^c^
3rd/4th degree perineal tear	24 (8.9%)	15 (9.2%)	0.90^c^
Menopausal state			
Premenopausal	40 (12.5%)	41 (21.8%)	**0.005** ^c^
Postmenopausal	281 (87.5%)	147 (78.2%)	
Duration of complaints in months	11 (3–24)	12 (3–36)	0.37^b^
Vaginal atrophy	100 (33.8%)	53 (30.5%)	0.46^c^
Prolapse stage			
II	141 (42.1%)	89 (43.6%)	0.73^c^
≥III	194 (57.9%)	115 (56.4%)	
PGIS score^d^			
I	44 (14.3%)	4 (2.1%)	**<0.001** ^b^
II	86 (28.0%)	25 (13.2%)	
III	135 (44.0%)	114 (60.3%)	
IV	42 (13.7%)	46 (24.3%)	
PFDI‐20 score^e^	60.6 (±37.3)	73.6 (±40.5)	**<0.001** ^a^
POPDI‐6	25.5 (±16.3)	29.7 (±16.9)	**0.006** ^a^
CRADI‐8	12.1 (±13.6)	16.2 (±15.1)	**0.002** ^a^
UDI‐6	22.8 (±18.2)	27.7 (±19.4)	**0.005** ^a^

*P* values in bold are significant. Percentages are based on the available data: 81–100%.

a Independent samples Student’s *t*‐test.

b Mann–Whitney *U*‐test.

c Chi‐square test.

d PGIS score: I (not severe), II (mild), III (moderate), IV (severe).

e The subscale scores vary between 0 and 100 points, and the total PFDI‐20 score varies between 0 and 300 points.

### Outcomes

Both primary and secondary outcomes are presented in Table 2. Data on types of interventions and adverse events are presented in Table S1. A statistically significantly higher proportion of women in the surgery group reported subjective improvement. At 24 months, the difference between the groups was 9.4% (95% CI 1.4–17.3%, *P* < 0.01) in favour of surgery. In addition, after correcting for differences in potential baseline confounding factors, women in the surgery group had 1.8 times higher odds (95% CI 1.0–3.6, *P* = 0.06) of successful improvement, compared with the pessary group. Regarding the PGI‐S, both groups reported statistically significant reductions in the severity of symptoms, although the proportion of women who reported less severe symptoms was significantly higher in the surgery group compared with the pessary group at 24 months (89.6 versus 64.5%).

**Table 2:** Primary and secondary outcomes

	Pessary	Surgery	Risk difference (95% CI; *P*)	Crude relative risk (95% CI; *P*)	Adjusted relative risk (95% CI; *P*)	*P*
PGI‐I: success^a^						
12 months	188/254 (74.0%)	144/171 (84.2%)	10.19 (2.52–17.87; **0.009**)	1.65 (1.10–2.46; **0.016**)	1.9 (1.1–3.3; **0.03**)	
24 months	180/242 (74.4%)	134/160 (83.8%)	9.37 (1.44–17.30; **<0.0001**)	1.57 (1.04–2.38; **0.030**)	1.8 (1.0–3.6; 0.06)	
PGI‐S: improved^b^						
12 months	154/247 (62.3%)^e^	140/164 (85.4%)^f^	23.02 (14.91–31.13; **<0.0001**)	1.37 (1.22–1.54; **<0.0001**)	1.38 (1.22–1.56; **<0.0001**)	
24 months	149/231 (64.5%)^e^	138/154 (89.6%)^f^	25.11 (17.28–32.94; **<0.0001**)	1.39 (1.24–1.55; **<0.0001**)	1.30 (1.18–1.45; **<0.0001**)	
			*P*‐value			
Switch to surgery or add. use of pessary	79 (23.6%)	8 (3.9%)				
Period of initial treatment, median days (IQR)	167 (78–514)	262.5 (159–615)	0.36^d^			
Discont. rate^c^ or reoperation		16 (7.8%)				
6 weeks	53 (16.6%)	1 (0.5%)				
12 months	84 (30.1%)	13 (6.3%)				
24 months	102 (40.6%)	2 (1.0%)				
Reasons for switch to surgery or reoperation						
Pessary expulsion	23 (29.1%)	n/a				
Inadequate symptom relief	16 (20.3%)	n/a				
Recurrence of prolapse	n/a	4 (2.0%)				
Discomfort/pain	14 (17.7%)	n/a				
Incontinence	10 (12.7%)	7 (3.4%)				
Excessive discharge	5 (6.3%)	n/a				
Dissatisfied about self‐management	4 (5.1%)	n/a				
Prefer surgery	3 (3.8%)	n/a				
Problems with sexual functioning	2 (2.5%)	n/a				
Decubitus	1 (1.3%)	n/a				
Unknown	1 (1.3%)	n/a				
Primary prolapse in untreated compartment	n/a	3 (1.5%)				
Small incision for dyspareunia	n/a	1 (0.5%)				
Small defect fornix posterior	n/a	1 (0.5%)				

*P* values in bold are significant.

a The PGI‐I questionnaire at 12 and 24 months was completed by 254 and 242 women in the pessary group and 171 and 160 women in the surgery group, respectively.

b The PGI‐S questionnaire at 12 and 24 months was answered by 247 and 231 women in the pessary group and 164 and 154 women in the surgery group, respectively.

c Based on available data, excluding physical loss of follow‐up and withdrawal of informed consent.

d Wilcoxon’s rank sum test.

e Within group a median decrease in PGI‐S score of 1.00 points (*P* < 0.001) between baseline and follow‐up.

f Within group a median decrease in PGI‐S score of 2.00 points (*P* < 0.001) between baseline and follow‐up.

Between baseline and the 24‐month follow‐up a total of 102 women (30.4%) stopped using a pessary and 79 women (23.6%) switched to surgery. The most common reasons for a switch to surgery were pessary expulsion and insufficient symptom relief. Not all women completed the follow‐up: in the worst‐case scenario, all women lost to follow‐up (including withdrawal of consent) stopped pessary therapy and 149 women (44.5%) continued pessary therapy at 24 months. In an optimistic scenario, 233 women (69.5%) continued pessary therapy at 24 months. Of the 43 women who did not return for physical follow‐up, 33 women (76.6%) completed the PGI‐I at 24 months and 28 (84.8%) of these women reported subjective improvement.

In the surgery group a total of 22 (10.8%) women underwent additional treatment because of recurrent prolapse and/or urinary incontinence (14 with re‐surgery and eight with additional pessary). An additional pessary was used seven times because of recurrent prolapse and once because of urge incontinence. Seven women (3.4%) were indicated for re‐surgery because of a recurrence of prolapse in either a treated or untreated compartment and seven women (3.4%) had re‐surgery because of stress urinary incontinence (SUI). Of these women, two needed a third intervention either because of SUI after re‐surgery for recurrent prolapse or because of a recurrent prolapse after re‐surgery for SUI.

**Figure 1:**
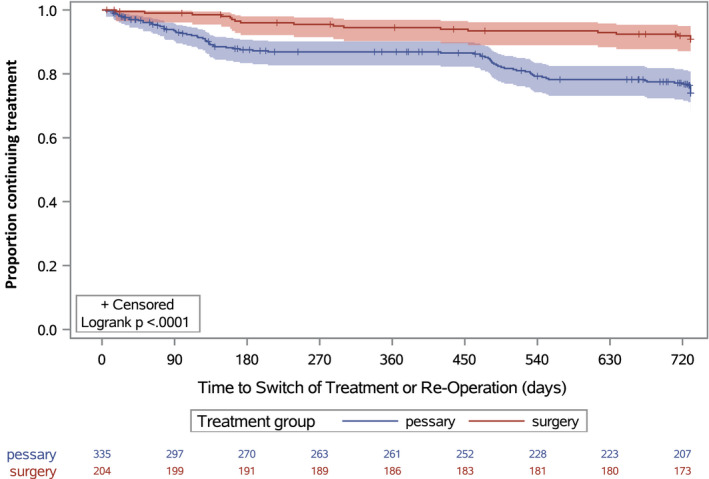
Kaplan–Meier plot for time to additional treatment or reoperation.

Women who had initially decided to undergo pessary therapy discontinued or needed additional treatment sooner than women who initially opted for surgery (Figure 1). The log‐rank test indicated significant differences between the two groups (*P* < 0.001). Censored subjects are shown on the Kaplan–Meier curve and indicate those who were lost to follow‐up.

The change in PFDI‐20 scores between baseline and the 24‐month follow‐up showed that both groups experienced a statistically significant improvement on all subscales and in total score (Table 3). Looking at the subscales of the PFDI‐20, using ES, we observed the following: the improvement on the POPDI‐6 scale was of high clinical relevance (ES > 0.8) for both interventions. With respect to the CRADI‐8 scale, the ES in both interventions showed a small clinical relevance (ES < 0.5). The UDI‐6 scale for the pessary group revealed weak clinical relevance (ES < 0.3), in contrast with the surgery group, which demonstrated moderate clinical relevance (ES > 0.6), indicating that the clinical relevance of the improvement is at least of moderate importance. This difference in clinical relevance was further analysed by examining the subscales of the UDI‐6.

**Table 3:** Outcomes of the pelvic floor distress inventory (PFDI‐20)

PFDI‐20 scales^a^	Pessary		Surgery		Mean difference in Δ between groups (95% CI)	*P*
	Δ (95% CI)	ES^b^	Δ (95% CI)	ES^b^		
Outcome 12 months^c^						
POPDI‐6	−18.0 (−20.0 to −15.8)^e^	1.00	−22.7 (−25.2 to −20.1)^e^	1.36	−4.7 (1.3–8.0)	**0.007** ^f^
CRADI‐8	−3.1 (−4.4 to −1.6)^e^	0.27	−6.7 (−8.7 to −4.7)^e^	0.50	−3.6 (1.3–6.0)	**0.003** ^f^
UDI‐6	−7.4 (−9.7 to −5.2)^e^	0.39	−13.7 (−16.6 to −10.7)^e^	0.71	−6.3 (2.6–10.0)	**0.001** ^f^
PFDI‐20	−28.5 (−33.1 to −23.9)^e^	0.76	−43.2 (−49.0 to −37.5)^e^	1.14	−14.7 (7.4–22.1)	**<0.001** ^f^
Outcome 24 months^d^						
POPDI‐6	−17.2 (−19.6 to −14.9)^e^	0.92	−23.1 (−25.7 to −20.5)^e^	1.40	−5.9 (2.3–9.5)	**0.001** ^f^
CRADI‐8	−2.4 (−3.9 to −0.9)^e^	0.20	−6.4 (−8.5 to −4.3)^e^	0.48	−4.0 (1.4–6.5)	**0.002** ^f^
UDI‐6	−6.2 (−8.9 to −3.5)^e^	0.29	−11.7 (−14.8–−8.7)^e^	0.61	−5.5 (1.4–9.6)	**0.008** ^f^
PFDI‐20	−26.3 (−31.5 to −21.1)^e^	0.65	−41.3 (−47.2 to −35.5)^e^	1.11	−15.0 (7.1–23.0)	**<0.001** ^f^

*P* values in bold are significant.

Δ refers to the change between baseline and follow‐up.

a The subscale scores vary between 0 and 100, the total PFDI‐20 score varies between 0 and 300. A negative change in score indicates an improvement.

b Effect size (Cohen’s *d*).

c At 12 months the questionnaire was sufficiently completed by 261 women in the pessary group and 170 women in the surgery group.

d At 24 months the questionnaire was sufficiently completed by 239 women in the pessary group and 159 women in the surgery group.

e Significance level <0.002 within group between baseline and follow‐up, tested with paired‐samples Student’s *t*‐test.

f Independent samples Student’s *t*‐test.

The UDI‐6 is composed of three subscales, involving SUI, obstructive and irritative symptoms.^40^ At 24 months the mean difference in improvement in score between groups on the SUI scale was 2.5 points (95% CI −2.7 to 7.8, *P* = 0.3). Regarding obstructive symptoms the mean difference in score was 6.0 points (95% CI 0.6–11.4, *P* = 0.03), and for irritative symptoms the mean difference in score was 7.5 points (95% CI 1.9–13.1, *P* < 0.01), all in favour of surgery.

Table S2 shows the results of the multivariable binomial regression for the three defined groups. A significantly higher proportion of women who had initial surgery considered themselves successfully improved, compared with women who primarily chose and retained a pessary. There was no additional benefit for women who underwent surgery after pessary therapy on the primary outcome.

## Discussion

### Main findings

This study shows that surgery, in comparison with pessary treatment, resulted in statistically significant more women reporting subjective improvement. One out of five women switched from pessary to surgery within 24 months. However, both interventions showed significant reductions in the presence and severity of prolapse symptoms, consistent with results of other comparative studies.^10^, ^22^, ^41^, ^42^ Surgery was more effective on secondary outcomes, as shown by significant reductions on the PGI‐S and PFDI‐20 scales. The favourable effect of surgery persisted after multivariable regression analysis.

### Strengths and limitations

In this cohort, patients were not randomised and differences in prognostic factors may have confounded our outcomes. The major advantage of an RCT is that randomisation effectively prevents confounding by design. However, many women decided not to participate in the RCT because of a strong preference in treatment choice. Inherently, women who did decide to participate in the RCT are also a selective group with potentially limited generalisability. In order to account for the differences at baseline between groups we adjusted outcomes in a multivariable analysis. Differences between surgery and pessary groups were found to be independent of baseline differences. Additionally, by allocating intervention based on shared decision making, our study reflects real clinical practice, which enhances the external validity of the findings.

Another limitation is that not all women completed follow‐up. In our optimistic scenario we would have a continuation rate of 69.5% at 24 months, which is comparable with other literature reporting continuation rates of between 64.0 and 75.4%.^18^, ^19^ Therefore, it is likely that we underestimated the continuation rate, assuming that women who did not return to the hospital remained satisfied with a pessary. This is supported by the fact that the majority of women who did not return for follow‐up reported successful improvement on the PGI‐I.

Analysis of the time to discontinuation of treatment or need for additional treatment, by means of the log‐rank test and Kaplan–Meier plot, relies on the assumption of an equal chance of observing the outcomes in patients censored as compared with those not censored. In this study, however, there is a larger proportion of women censored in the pessary group than in the surgery group. A possible reason could be dissatisfaction with symptom relief leading to dropout. In that case it is possible that the loss to follow‐up reflects a competing risk.

As 23.6% crossed over from pessary to surgery we might have overestimated the effect of pessary treatment. To address this problem, a multivariable binomial regression was performed for the continuing pessary users, for the switchers from pessary to surgery and for the surgery group. This showed no favourable effect of additional surgery in the pessary group but did confirm the significantly better treatment effect of surgery over pessary.

Strengths of this study include the participation of 22 hospitals across the country. This widespread distribution of participating patients and gynaecologists increases the generalisability of our findings.

Another strength was the possibility of performing a reliable multivariable regression with a large sample size of 539 women. The multivariable binomial regression depends on maximum‐likelihood estimation, and the reliability of estimates declines when observed outcomes or predictors are sparse.^37^ To our knowledge, this is one of the few studies with such a large sample size.

Furthermore, we used patient‐reported validated outcomes because objective measures like prolapse stage have been shown to underestimate the degree of subjective symptom‐related distress experienced by women.^43^ A third strength is the long‐term follow‐up of 24 months, which is recommended by the NICE guidelines.^24^

### Interpretation

In line with other studies, women opting for surgery are significantly younger, have a higher BMI and experience more bothersome symptoms at baseline.^10^, ^20^, ^44^ Apparently, women who experience more bothersome symptoms are more likely to choose surgery. Compared with women who chose to use a pessary, women who opted for surgery had greater improvements in their prolapse, bowel and urinary distress symptoms, and more often reported a subjective improvement in their quality of life. However, women should be counselled that approximately one out of 13 will undergo reoperation, which is in line with previous findings.^45^ The main reasons for reoperation are recurrence of prolapse and/or SUI. In order to make a well‐balanced decision, it is important to emphasise that although a pessary is less effective than surgery it does constitute an effective treatment option and should be discussed with women considering surgery.

Focusing on the subscales of the PFDI‐20 it is clear that both therapies relieved POP symptoms to a clinically relevant level, as shown by the effect sizes on the POPDI‐6 scale. With respect to the CRADI‐8 subscale we found a statistically significant improvement in both groups. This is in contrast with a previous study reporting that the CRADI‐8 subscale was not significantly improved in the pessary group.^35^ A possible explanation is that our large sample size has contributed to detecting a statistically significant difference that is not, by definition, clinically relevant. The ES for the difference in CRADI‐8 subscale score between baseline and the 24‐month follow‐up in the pessary group was only 0.2, indicating limited clinical significance. A possible explanation for the lack of a clinical significant effect on bowel symptoms can be related to the fact that a pessary provides limited support to the posterior vaginal wall.^46^ This is supported by the fact that the surgery group reported significantly more symptom reduction on the CRADI‐8 subscale, although the effect size is only moderate. An alternative explanation is that bowel symptoms correlate poorly with the side and stage of the POP, as demonstrated previously.^47^

With respect to the UDI‐6 scale, the surgery group showed statistically significant more reduction in urinary symptoms. Using the ES, the clinical effect was of moderate importance, as compared with the pessary group showing a limited clinical effect. Focusing on the subscales of the UDI‐6, the surgery group showed a statistically significantly greater improvement on obstructive and irritative symptoms. A possible explanation is that there is a causal relationship between overactive bladder symptoms (OAB) and POP.^48^ One hypothesis is that POP induces symptoms of OAB, detrusor overactivity and voiding dysfunction as a result of urethral kinking and urethral compression.^49^, ^50^ It might be that surgery provides a better anatomical outcome because it reduces urethral mobility and urethral kinking.^51^

Another hypothesis is that distention of the bladder, and thereby the prolapsing of the bladder trigone, causing a nearby closed bladder neck, cause ischaemia and hypoxia of the bladder wall.^52^ A pathological response might be partial denervation of the autonomic nerve supply to the detrusor muscle.^52^ Another study found that in overactive bladders there was a supersensitised reaction to the main neurotransmitter of the bladder and reduced nerve‐mediated responses.^53^

Our hypothesis is that a pessary still applies pressure to the bladder trigone, inducing the same response.

## Conclusion

In case of predominant prolapse symptoms, both therapies showed a clinically important improvement and can be advised as a primary treatment. When bothersome micturition and defecation symptoms coexist, surgery is more effective. Women should be counselled that after 24 months 73% of women who continued pessary therapy reported a successful improvement, as compared with 84% after surgery. One out of five women who started with pessary therapy switched to surgery within 24 months because of side effects or insufficient symptom relief.

### Disclosure of interests

None declared. Completed disclosure of interests form available to view online as supporting information.

### Contribution to authorship

LRV was the main author of this article. AV was the project leader, secretary and grant applicant for this study. CHV was the main applicant of this study. JPWR was the doctoral supervisor of LRV. ALM was involved as a co‐project leader. ALJ was involved as a co‐applicant. AV, CHV, JPWR and ALM were all involved in the inclusion of the participants. RGD was involved in the statistical analysis of our data. All contributing authors were involved in revising the article and all gave approval for the final version to be published.

### Details of ethics approval

This study was conducted according to the principles of the Declaration of Helsinki (version 10, October 2013) and in accordance with the Medical Research Involving Human Subjects Act (WMO) and other guidelines, regulations and acts. This study was approved by the Medical Research Ethics Committee (MREC), or Medisch Ethische Toetsing Commissie (METC), in Dutch. The date of approval was 22 September 2015 and the METC protocol number is 14‐533/M. The METC required a Data Safety Monitoring Board (DSMB). The monitoring was coordinated by the Dutch consortium and was executed by a qualified intern monitor. This person was not involved in the design and output of this research. The frequency of checking was every year. The investigator will submit a summary of the progress of the trial to the accredited METC once a year.

### Funding

This study was funded by ZonMW, a Dutch governmental healthcare organisation. This organisation finances healthcare research and stimulates the use of the knowledge developed to improve care and health. This study was funded on 26 June 2014 (project no. 837002525).

### Acknowledgements

PEOPLE group: J. van Bavel, M.Y. Bongers, K. Bos, A.M.W. Broekman, J.P. de Bruin, V. Dietz, H.W.F. van Eijndhoven, R. Hakvoort, E. Janszen, K.B. Kluivers, G. Link, D. Massop‐Helmink, A.L. Milani, J.P.W.R. Roovers, R.P. Schellart, F.M. Sikkema, S. Stekelenburg‐de Vos, A. van der Ster, C.H. van der Vaart, L.R. van der Vaart, M.M.A. Vernooij, L. van der Voet, A. Vollebregt, M. Weemhoff.

## Supporting information


**Figure S1**. Flow chart for the inclusion and follow‐up of patients.

**Table S1**. Types of interventions and adverse events.
**Table S2**. Analysis of the primary outcome (PGI‐I) for the three defined groups.

Supplementary Material

Supplementary Material

Supplementary Material

Supplementary Material

Supplementary Material

Supplementary Material

Supplementary Material

## Data Availability

The data that support the findings of this study are available from the corresponding author, upon reasonable request.
