# Supplementary material for: Pessary or surgery for a symptomatic pelvic organ prolapse: the PEOPLE study, a multicentre prospective cohort study
Source: BJOG. 2021 Oct 28;129(5):820–9. doi: 10.1111/1471-0528.16950 (PMC9298049; doi:10.1111/1471-0528.16950)
Supplement: Supplementary file 1 — Figure S1. Flow chart for the inclusion and follow‐up of patients. [file BJO-129-820-s009.docx]

554 women were asked to participate

- 9 did not provide informed consent

- 3 informed consent went missing

- 1 excluded due to protocol violation

- 1 excluded due to prolapse stage I

- 1 excluded due to missing prolapse stage

335 included in the pessary group

- 43 (12.8%) withdrew consent before 24- months follow-up

- 50 (14.9%) loss of follow-up ¶

- 10 (4.9%) withdrew consent before 24- months follow-up

- 34 (16.7%) loss of follow-up ¶

242 (72.2%) included in the 24-months analysis of the primary outcome

**Figure 1. Inclusion and follow-up**

539 women were included

204 included in the surgery group

160 (78.4%) included in the 24-months analysis of the primary outcome

¶ The loss of follow-up represents those women who did not complete the PGI-I (primary outcome) at 24-months follow-up.
