# Supplementary material for: Pessary or surgery for a symptomatic pelvic organ prolapse: the PEOPLE study, a multicentre prospective cohort study
Source: BJOG. 2021 Oct 28;129(5):820–9. doi: 10.1111/1471-0528.16950 (PMC9298049; doi:10.1111/1471-0528.16950)
Supplement: Supplementary file 2 — Table S1. Types of interventions and adverse events. Table S2. Analysis of the primary outcome (PGI‐I) for the three defined groups. [file BJO-129-820-s004.docx]

| **Table S1. Types of interventions and adverse events.** | | | |
| --- | --- | --- | --- |
|  | Pessary group  N = 335 (%) |  | Surgery group  N = 204 (%) |
| Type of pessary |  | Type of surgery* |  |
| Supportive | 325 (97.0) | ACR | 21 (10.3) |
| Occlusive | 10 (3.0) | PCR | 26 (12.7) |
|  |  | SSH | 5 (2.5) |
|  |  | MF | 15 (7.4) |
|  |  | VH | 7 (3.4) |
|  |  | LC | 1 (0.5) |
|  |  | ACR + PCR | 11 (5.4) |
|  |  | ACR + PCR + SSH | 14 (6.9) |
|  |  | ACR + PCR + MF | 5 (2.5) |
|  |  | ACR + PCR + VH | 13 (6.4) |
|  |  | ACR + SSH | 52 (25.5) |
|  |  | ACR + MF | 20 (9.8) |
|  |  | ACR + VH | 5 (2.5) |
|  |  | ACR + SSH + TS | 1 (0.5) |
|  |  | PCR + SSH | 2 (1.0) |
|  |  | PCR + MF | 3 (1.5) |
|  |  | PCR + VH | 2 (1.0) |
|  |  | PCR + VH + LC | 1 (0.5) |
| Adverse events ¶ |  | Adverse events |  |
| Discomfort/ pain | 122 (36.4) | Urinary bladder retention | 15 (7.4) |
| Excessive discharge | 78 (23.3) | Urinary tract infection | 9 (4.4) |
| Urinary stress-incontinence | 48 (14.3) | Blood loss > 500cc | 4 (2.0) |
| Decubitus | 44 (13.1) | Transfusion due to blood loss | 1 (0.5) |
| Blood loss | 35 (10.4) | Hematoma | 1 (0.5) |
| (Recurrent) urinary tract infection | 7 (2.1) | Bladder lesion | 1 (0.5) |
| Vaginal infection | 6 (1.8) | Vocal cord damage due to intubation | 1 (0.5) |
| Vaginal atrophy | 5 (1.5) | Vulvar pain | 1 (0.5) |
| Urge-incontinence | 2 (0.6) | Hospital acquired pneumonia | 1 (0.5) |
| Dysfunctional voiding | 2 (0.6) | Admission for 7 days due to post-operative fever | 1 (0.5) |
| Mixed incontinence | 1 (0.3) | Synechiae between anterior and posterior vaginal wall | 1 (0.5) |
|  |  | Vaginal wound infection | 1 (0.5) |
| ***ACR** Anterior colporrhaphy, **PCR** Posterior colporrhaphy, **SSH** Sacrospinous hysteropexy, **MF** Modified Manchester-Fothergill, **VH** Vaginal hysterectomy, **LC** Laparoscopic sacrocolpopexy **TS** Transobturator sling.  ¶ A total of 350 adverse events happened in 197 women (58.8%) | | | |

\

| **Table S2. Analysis of the primary outcome (PGI-I) for the 3 defined groups** | | | | | | |  |  |
| --- | --- | --- | --- | --- | --- | --- | --- | --- |
|  | Pessary only  N (%) | Surgery after pessary  N (%) | Surgery only  N (%) | *p*-value | | | Adjusted OR (95%CI, *p-value*) | Adjusted OR (95%CI, *p-value*) |
| **12 months follow-up ¶** | | | | Group 1 vs. 2 | Group 2 vs. 3 | Group 1 vs. 3 | Group 1 vs 2 | Group 1 vs 3 |
| success/total n. (%) | 148/203 (72.9) | 40/51 (78.4) | 144/171 (84.2) | 0.42^1^ | 0.34^1^ | **0.008^1^** | 1.4  (0.6 – 3.3, 0.38) | 2.0  (1.1 – 3.7, **0.02**) |
| **24 months follow-up** ¤ | | | | | | |  |  |
| success/total n. (%) | 128/175(73.1) | 52/67 (77.6) | 134/160 (83.8) | 0.48^1^ | 0.27^1^ | **0.02^1^** | 1.8  (0.8 – 4.0, 0.13) | 2.1  (1.1 – 3.9, **0.02**) |
| *p* values in bold are significant.  **¶** The PGI-I question at 12-months was answered by 203 who only had a pessary, 51 who had surgery after pessary and 171 women who only had surgery.  ¤ The PGI-I question at 24-months was answered by 175 who only had a pessary, 67 who had surgery after pessary and 160 women who only had surgery.  Groups: 1. Pessary only; 2. Surgery after pessary; 3. Surgery only.  1.Chi-square test. | | | | | | | | |
